# A sharing oocyte donation program: a 15-year cohort stud

**DOI:** 10.5935/1518-0557.20220056

**Published:** 2023

**Authors:** Talita Colombo, Marta Ribeiro Hentschke, Mariangela Badalotti, Ariane Tieko Frare Kira, Isadora Badalotti Telöken, Vanessa Devens Trindade, Victoria Campos Dornelles, Alvaro Petracco, Eliana Márcia Wendland

**Affiliations:** 1 Federal University of Health Sciences of Porto Alegre (UFCSPA), Porto Alegre, Brazil; 2 Fertilitat Reproductive Medicine Center, Porto Alegre, Brazil; 3 School of Medicine, Pontifical Catholic University of Rio Grande do Sul, Porto Alegre, Brazil

**Keywords:** oocyte donation, IVF, reproductive outcomes

## Abstract

**Objective:**

To evaluate clinical and laboratory outcomes of oocyte donation cycles and compare the results from donors and recipients.

**Methods:**

A retrospective cohort study was conducted at a reproductive medicine center. A 586 first fresh oocyte donation cycles, performed from 01/2002 to 12/2017 were included. The outcomes of 290 cycles from donors and 296 from recipients, resulting in 473 fresh embryo transfers, were analyzed. The oocyte division was equally made, whereas, at an odd amount, the donor always had a preference. The data were collected from an electronic database, and analyzed using Chi-square test, Fisher’s exact test, Mann-Whitney U-test or Student t-test depending on the data distribution, and multivariate logistic regression, considering *p*<0.05.

**Results:**

The main results comparing donor and recipient, were, respectively: fertilization rate (72.0±21.4 *vs*. 74.6±24.2, *p*<0.001), implantation rate (46.2% *vs*. 48.5%, *p*=0.67); clinical pregnancy rate (41.9% *vs*. 37.7%, *p*=0.39), live birth rates by transfer (33.3 *vs*. 37.7, *p*=0.54).

**Conclusions:**

Oocyte donation is often the way donors can access in vitro fertilization, and for recipients seems to be a good option for pregnancy. Demographic and clinical characteristics have a secondary role in oocyte donors under 35 years and patient without comorbidities under 50 years and were not associated with pregnancy outcomes, emphasizing the power of oocyte quality on the success of intracytoplasmic sperm injection treatment. An oocyte-sharing program that offers good and comparable results is fair and worth being encouraged.

## INTRODUCTION

*In vitro* fertilization (IVF) is an assisted reproduction technique (ART) in which the fertilization process is performed at a laboratory, and the embryos selected are transferred to the woman’s uterus (Teede *et al*., 2018). The possibility of extracorporeal extraction eventually led to a wide range of ART procedures, including the donation of oocytes from one woman to another one. In an ART treatment, oocyte donation (OD) is a process in which a woman allows her oocytes to be collected after ovarian stimulation (donor), which can be used for another infertile woman (recipient) to get pregnant by IVF.

The first report of a child born through this procedure occurred in Australia in 1983 (Trounson *et al*., 1983). Since then, the demand for this treatment modality has been increasing substantially, and today, ARTs involving third-party oocytes (unfertilized human egg cells, ovules, or female gametes) account for nearly 10% of all ART cycles in the United States (Melnick & Rosenwaks, 2018). The American Society for Reproductive Medicine (ASRM) data has shown that in the United States, more than 7.800 cycles of IVF with OD were performed in 2016 (Practice Committee of the American Society for Reproductive Medicine & the Practice Committee for the Society for Assisted Reproductive Technology, 2021). In Europe, 39,000 ART cycles with third-party oocytes are undertaken annually (European IVF-monitoring Consortium, 2017), and in 2018, there were 14,435 embryo transfers from both fresh and frozen-thawed oocyte donation in Latin America. Currently, OD represents 18.1% of all ART procedures in this region (Zegers-Hochschild et al., 2021). This method became the standard treatment for infertility related to premature ovarian insufficiency and is also indicated for women who have failed repeated IVF treatments, women with age-related infertility, hypergonadotropic hypogonadism, and those who are known to be affected by or to be the carrier of a significant genetic defect, or who have a family history of a condition for which the carrier status cannot be determined (Practice Committee of the American Society for Reproductive Medicine & the Practice Committee for the Society for Assisted Reproductive Technology, 2021). The legal status and compensation models of OD vary significantly between countries: in Germany and Japan, it is prohibited; in France, Greece, Hungary, Italy, Poland, Portugal, Slovenia, and Spain, it is allowed with anonymity, and in Austria, Finland, Netherlands, Sweden, and in the United Kingdom it is non-anonymous. In the United States, South Africa, India, and Cyprus, it is allowed and financially compensated (Bergmann, 2011; CIV10.ES, 2022). Spain is worldwide famous for oocyte banks and currently performs half of the 39,000 cycles with third-party oocytes annually in Europe (Degli Esposti & Pavone, 2019). In Brazil, until 2017, only patients who had an indication for ART could be included in the OD program, which consists of donations by both parties involved: one woman contributes with the oocytes, and the other with the funding of the treatment (OVODON Program). Nowadays, a voluntary and non-commercial OD without an IVF procedure associated, or the use of oocyte banks is also possible.

This treatment modality can lead to live birth rates upwards of 50% per cycle (European IVF-monitoring Consortium, 2017). Some factors have been reported as predictors of birth in autologous cycles, including maternal age, body mass index (BMI), ethnicity, antral follicle count (AFC), prior parity, and cause and time of infertility (Practice Committee of the American Society for Reproductive Medicine & the Practice Committee for the Society for Assisted Reproductive Technology, 2020). Elucidative factors that can optimize results in OD cycles is a crescent and relevant demand. Also, encouraging women to choose this method when indicated and showing its benefits are of great importance. Thus, the aim of this study was to evaluate clinical and laboratory factors of OD cycles and compare the OD results from donors and recipients.

## MATERIAL AND METHODS

### Study Design

Retrospective cohort study.

### Patient sample, study period, and location

We evaluated the outcomes of 586 first OD cycles performed between January 2002 and December 2017 at a private reproductive medicine center in Brazil, following the current Brazilian regulation (CFM, 2017) and standard clinical practice. All oocyte donors were included in the oocyte-sharing program voluntarily, anonymously, and without financial compensation. They were younger than 35 years old, had a good ovarian reserve, a normal clinical and gynecology evaluation, presented a normal karyotype, and negative serological tests (human immunodeficiency virus, HIV, types 1 and 2; human T cell lymphotropic virus, HTLV, types 1 and 2; hepatitis B virus, HBV; hepatitis C virus, HCV; rubella virus; human cytomegalovirus, CMV; *Toxoplasma gondii, T. gondii*; nontreponemal test for syphilis, VDRL; and, since 2015, Zika virus, ZIKV). The recipients were also included in the oocyte-sharing program voluntarily and anonymously after a psychological evaluation. They were less than 50 years old and had a normal clinical and gynecology evaluation and negative serological tests.

### Study variables

Data collection had four components: baseline characteristic data, ovarian stimulation data, embryo development data, and pregnancy outcomes. The detailed list of included variables is available in the results tables.

### Ovarian Stimulation (Donors)

Donors underwent controlled ovarian stimulation with gonadotrophins in one of two protocols: a long gonadotropin hormone-releasing hormone (GnRH) agonist or a GnRH antagonist protocol. The trigger was done with human chorionic gonadotropin (hCG) and was performed when at least three follicles reached a diameter of 17 mm. Oocyte retrieval was performed 35 hours after hCG.

### Oocyte division and insemination

According to the rules of our OD program, the total number of mature (metaphase II) oocytes was equally divided between groups, whereas at an odd amount, the donor always had a preference. Between 2-4 hours after retrieval, the oocytes were inseminated.

### Embryo Culture

Embryos were cultured in the cleavage medium for the first 72 hours after fertilization and subsequently in the blastocyst medium until day 5. The day of embryo transfer was determined based on the quantity and quality of embryos on either day 3 (D3, cleavage stage) or day 5 (D5, blastocyst stage). Cleavage-stage embryos were defined as good quality (grade 1-2), and day 5 blastocysts were graded according to size, inner cell mass, and trophectoderm development. Good-quality blastocysts were classified according to the Gardner grade (ESHRE Special Interest Group of Embryology & Alpha Scientists in Reproductive Medicine, 2017).

### Recipients’ endometrial preparation

The periods of donor and recipient are synchronized by hormonal contraceptive pills to allow recipients to receive fresh embryos. The endometrium preparation was made with estrogen (4-8 mg estradiol valerate daily) administered until a thick trilaminar endometrium (at minimum 8 mm) was achieved. In women with inadequate response to oral estrogen supplements, transdermal estrogen was added (6 mg estradiol daily). On the day of oocyte retrieval, vaginal progesterone (600 mg micronized progesterone daily) was initiated.

### Donors’ luteal phase

On oocyte retrieval day, the donors started vaginal progesterone (600 mg micronized progesterone daily).

### Outcomes - Defining the variables

Fertilization rate was defined as the proportion of injected oocytes with two pronuclei (2PN) 16-18 hours after injection. Implantation rate was defined as the number of gestational sacs observed divided by the number of embryos (cleavage-stage or blastocysts) transferred (ESHRE Special Interest Group of Embryology & Alpha Scientists in Reproductive Medicine, 2017). Clinical outcomes were evaluated as biochemical or clinical pregnancies. Biochemical pregnancy was defined as positive serum Beta-hCG test 12 days after blastocyst transfer or 14 days after cleavage-stage embryo transfer. Clinical pregnancy was defined as the presence of an intrauterine fetal heartbeat on the ultrasound. Clinical pregnancy outcomes were further categorized as miscarriage (spontaneous arrest of pregnancy before 20 weeks of gestation), ectopic pregnancy (embryo implants somewhere other than the uterus), stillbirth (intrauterine fetal death after 20 weeks of gestation), or live birth. Live birth per cycle was calculated by birth after an embryo transfer.

### Statistical analysis

Preliminary exploratory data analysis was used to evaluate variable distributions and to assess relations among key variables. Welch unequal variances t-test was used to test for significant differences between continuous variables. Multivariate logistic regression analyses were performed to assess the association between individual donor and recipient characteristics and clinical pregnancy and live birth rates. Candidate variables for the multivariate model were selected according to the log-likelihood test. Variables with a *p*<20 in the bivariate model were tested in the multivariate model, according to Hosmer & Lemeshow (2000). Quantitative variables were presented as mean ± standard deviation (SD) or median and interquartile range (IQR) as appropriate. Mann-Whitney U-test and Student t-test were used depending on the data distribution. For categorical variables, we used percentages and applied the Chi-square test or Fisher’s exact test, considering *p*<0.05. Statistical tests were conducted using the Statistical Package for Social Sciences version 21 (SPSS 21.0) for Windows.

### Ethical Issues

According to the Brazilian ethical rules, matching between donors and recipients considering phenotypic and health characteristics and identities were made by medical and psychological staff and kept confidential. The reproductive medicine center’s psychologist evaluated both oocyte donors and recipients separately. All data were collected following the principles of the Ethical Committee Resolution 466/2012. All authors signed a data compromise and confidentiality term for collecting data. Institutional Research and Ethics Board of the Federal University of Health Sciences of Porto Alegre (UFCSPA) approved the study at Number 3.108.739.

## RESULTS

**Figure 1:**
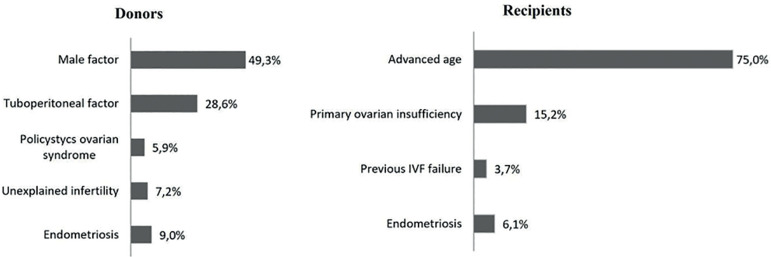
Main diagnosis of infertility in oocyte-donors and oocyte-recipients between 2002-2017.

In 586 consecutive donor oocyte-recipient cycles, there were 473 fresh embryo transfers. The main causes of infertility are presented in Figure 1, and the demographic and clinical characteristics of donors and recipients are presented in Table 1.

**Table 1:** Demographic and clinical characteristics of oocyte donors and recipients.

	Donors (n=290)	Recipients (n=296)	*p*
Female age, years, mean±SD	30.6±3.3	43.1±4.9	<0.001^*^
Male age, years, mean±SD	35.3±6.1	42.9±6.6	<0.001^*^
Type of infertility, n (%) Primary Secondary	255 (87.9) 35 (12.1)	225 (76.1) 71 (23.9)	<0.001^‡^
Previous parity, n (%)	17 (5.9)	38 (12.8)	0.006^‡^
Previous spontaneous abortion, n (%)	31 (10.6)	72 (24.3)	<0.001^‡^
Previous ectopic pregnancy, n	7 (2.4)	7 (2.3)	1.000^‡^
BMI, kg/m^2^, mean±SD	23.9±3.5	23.3±3.3	0.054^*^
Basal FSH, mUI/mL, median (25^th^-75^th^)	5.3 (3.6-8.9)^§^	36.1 (21.2-76.9)^||^	<0.001^†^
AFN, median (25^th^-75^th^)	18 (13-22)	0 (0-1)	<0.001^†^

BMI. Body mass index; FSH. follicle-stimulating hormone; AFN. Antral follicle count.

* Student t-test;

† Mann-Whitney U test;

‡ Pearson chi-square test.

§ n=36

|| n=116.

The main results comparing donor and recipient was, respectively, female age (years), 30.6±3.3 *vs*. 43.4±4.9, *p*<0.001; male age (years), 35±6.1 *vs*. 42.9±6.6, *p*<000.1; percentage of primary infertility, 87.9% *vs*. 76.1.1%, *p*<000.1; percentage of previous abortions, 10.6% *vs*. 24,3%, *p*<000.1. Comparing the age of recipients who became pregnant versus those who did not, the following result was found, respectively 42.5±4.9 *versus* 43.6±4.7, *p*=0.058. Cycle and laboratory characteristics are presented in Table 2.

**Table 2:** Cycle characteristics for oocyte donors and recipients.

	Donors (n=290)	Recipients (n=296)	*p*
Gonadotropin type, n (%) HP-FSH rFSH hMG rFSH+hMG	18 (6.3) 132 (45.5) 33 (11.5) 107 (37.7)	NA NA NA NA	
Stimulation protocol, n (%) Antagonist Agonist	210 (72.4) 80 (27.6)	NA	
Oocyte aspirated, median (25^th^-75^th^)	14 (10-21)	NA	
Oocytes mature, median (25^th^-75^th^)	11 (8-16)	NA	
Oocytes mature after donation/reception, median (25^th^-75^th^)	6 (4-8)	5 (3-7)	
Oocyte fertilized, median (25^th^-75^th^)	4 (3-6)	4 (3.6)	0.953^†^
Endometrial thickness (mm), mean±SD	11.0±2.0	10.2±1.9	<0.001^*^
Sperm origin, n (%) Ejaculated Not ejaculated -Bank -PESA -TESA	242 (83.4) 16 (5.5) 14 (4.8) 18 (6.2)	281 (94.9) 7 (2.4) 4 (1.3) 4 (1.3)	<0.001^‡^ 0.095^‡^
Fertilization rate, mean±SD	72.0±21.4	74.6±24.2	<0.001^*^
Good quality D3 embryo, median (25^th^-75^th^)	2 (2-3)	2 (2-4)	0.446^†^
D5 blastocyst, median (25^th^-75^th^)	3(2-4)	3(2-4)	0.128^†^

FSH. follicle-stimulating hormone; HP-FSH. Highly purified FSH; rFSH. Recombinant FSH; hMG. human menopausal gonadotropin; PESA. Percutaneous epididymal sperm aspiration; TESA. testicular sperm extraction. D3. Day 3 of embryo development; D5 Day 5 of embryo development.

* Student t-test;

‡ Mann-Whitney U test;

† Pearson chi-square test.

**Figure 2:**
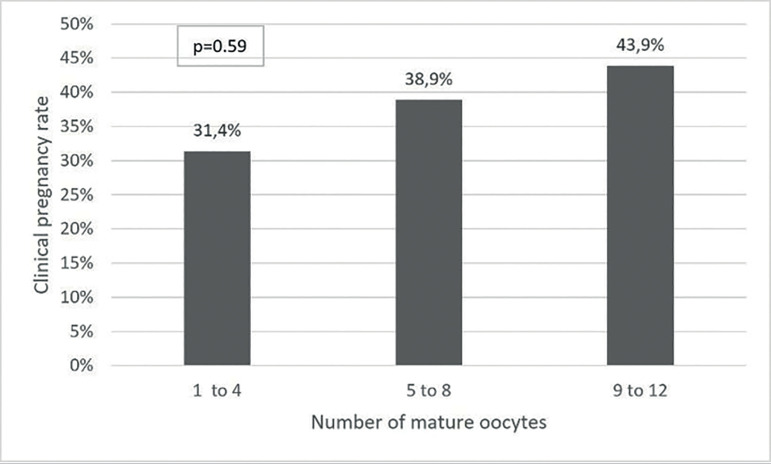
Rates of clinical pregnancy in recipients by number of oocytes obtained, analysis by logistic regression.

The main gonadotropin type used was FSHr (45.5%), followed by combined FSHr-hMG (37.7%). The use of ejaculated sperm was higher in recipients than in donors, that more frequently needed semen from a bank, PESA or TESA. (16.7 *vs*. 7.7, *p*<000.1). The outcomes in fresh OD cycles with fresh embryo transfer are shown in Table 3. Comparing donor and recipient, respectively, clinical pregnancy, 41.9% *vs*. 37.7%, *p*=0.39, and live birth rates per transfer, 33.3% *vs*. 27.1, *p*=0.54 had no statistically significant difference between groups. Finally, the incidence of clinical pregnancy in recipients by the number of oocytes obtained is shown in Figure 2. When a bivariate logistic regression was performed, demographic, hormonal, cycle, and laboratory characteristics did not differ between donors and recipients with or without clinical pregnancies.

**Table 3:** Outcomes in fresh oocyte donation cycles with fresh embryo transfer.

	Donors (n=234)	Recipients (n=239)	*P*	General
Transferred embryos, mean±SD	2.0±0.7	2.2±0.9	0.003^*^	2.1±0.8
Implantation rate, %	108/234 (46.2)	116/239 (48.5)	0.670^‡^	224/473 (47.4)
Biochemical pregnancy, %	10/234 (4.3)	26/239 (10.9)	0.011^‡^	36/473 (7.6)
Clinical pregnancy, %	98/234 (41.9)	90/239 (37.7)	0.398^‡^	188/473 (39.7)
Live birth, %	78/234 (33.3)	65/239 (27.1)	0.548^‡^	143/473 (30.2)
Multiple birth rate, %	20/234 (8.5)	18/239 (7.5)	0.931^‡^	38/473 (8.0)
Miscarriage’s rate, %	16/98 (16.3)	12/90 (13.3)	0.792^‡^	28/188 (14.9)
Ectopic pregnancy, %	6/234 (2.6)	5/23 8 (2.1)	0.982^‡^	11/473 (2.3)

* Student t-test;

‡ Pearson Chi-square test.

## DISCUSSION

This study aimed to evaluate clinical and laboratory outcomes of OD cycles and compare the results from donors and recipients. It is important to highlight that the clinical pregnancy and live birth rates were similar between donor and recipients, respectively (clinical pregnancy 41.9% *vs*. 37.7%, *p*=0.39, live birth rates per transfer, 33.3% *vs*. 27.1%, *p*=0.54), which already brings us an answer to the study question, showing that the program of egg donation is of paramount importance within the possibilities of ART.

The variables analyzed are discussed below.

### Demographic and clinical characteristics

In this study, no statistically significant differences were observed in demographic characteristics between donors and recipients in cycles that resulted or not in live birth, in agreement with Braga *et al.* (2020) recent publication which shows the lack of correlation between donor characteristics and cycles outcomes. However, it was possible to observe a tendency of higher rates of primary infertility in donors and higher rates of previous miscarriage in recipients. It was also found a possible relation between OD and the IVF programs access in order to achieve pregnancy. Besides that, previous miscarriage was more frequent in the recipients that have the advanced age as the main cause of infertility. In this group of patients OD may be the only option for pregnancy.

#### Oocyte donor age

Donors’ age was one of the most important donors’ characteristics, as oocyte age is one of the primary contributors to IVF outcomes. Younger donors usually are preferred to older ones since female fertility declines with age (Hipp *et al*., 2020). Hogan *et al*. (2019), in a retrospective cohort study, identified that the mean age of Australian oocyte donors was 33.7 years old, statistically significantly older than the average age of donors in studies from Europe and the United States, 27.4 years and 28 years old, respectively. In 2.919 fresh and frozen embryo transfer cycles, they found that recipients with donors aged <30 years had 44,7% cumulative live births (CLB), recipients with donors aged between 30-34 had 43.3% CLB, recipients with donors aged between 35-39 years had 31.0% CLB, and recipients with donors aged > 40 years had 10.5% CLB (Hogan *et al*., 2019). Conversely, Humphries *et al*. recently published a study regarding oocyte donors’ age and IVF outcomes, revealing that younger donor age does not necessarily correlate with greater treatment success in OD cycles in patients younger than 30 years; though not statistically significant, this study shows that cycles using donors <25 years were less likely to result in clinical pregnancy and live birth compared with cycles using donors 25 to <30 years, independently of recipient age, and despite similar numbers of oocytes retrieved and similar fertilization success. These data are consistent with reported data by Wu *et al.* (2012), in which women aged <25 had less favorable IVF treatment outcomes, such as clinical pregnancy and live birth rates, compared to women aged 25-35, due to higher prevalence of aneuploidy.

A retrospective cohort study from 2008 to 2015 of 350 oocyte donors who underwent a total of 553 ovarian stimulations and oocyte retrievals for a donor oocyte bank also found no associations between donor oocyte yield and probability of live birth, adjusting for donor age, BMI, race/ethnicity, and retrieval year (Hipp et al., 2020). In our study, by bivariate logistic regression, there was not an association between donors’ age and clinical pregnancy rates in recipients.

### Laboratory variables

The main finding of this long-time retrospective cohort was the similar laboratory evolution between donors and recipients.

#### Number of retrieved and mature oocytes

The number of retrieved and mature oocytes are an often variable used associated with live birth in studies considering autologous cycles in assisted reproduction techniques. In this study, the recipients group presented an increased rate of clinical pregnancy per retrieved oocytes, and, although this finding was not statistically significant, is similar to other studies when considering autologous cycles, such as Magnusson *et al*. (2018) that found a significant increase in the cumulative birth rate per retrieved oocytes (aOR 1,064, 95% CI: 1,061; 1,067). Furthermore, in fresh cycles analyzed by Magnusson et al. (2018), the rate of live births increased when 11 or more oocytes were retrieved, and the cumulative aspiration rate (including fresh and frozen cycles) reached 45.8% when about 20 oocytes were aspirated. Similarly, the multivariate analysis performed by Shavit *et al*. (2019) showed a correlation between birth rates in single embryo transfer and the number of aspirated oocytes in fresh cycles. In the same way, Hariton *et al.* (2017) observed that the relative risk of live birth was higher in cycles with >10 total oocytes retrieved and >10 mature oocytes retrieved, even after accounting for confounders of interest in a multivariable regression model. In another way, in OD cycles, there was no association between a higher number of retrieved oocytes and increased incidence of live births compared to autologous cycles (Baker *et al*., 2010). It is important to consider that in our study, the total number of mature oocytes was divided between donors and recipients, and we only analyzed results in single fresh cycles; thus, cumulative pregnancy assessment was not possible to be evaluated.

#### Fertilization rates and embryos culture

The groups had similar number of D3 good-quality embryos and blastocyst, as well as the proportions of fresh embryos transfers. Also, donors and recipients had similar implantation rates, clinical pregnancy, and live birth rates per transfer. The fertilization rates were different between groups, but withou clinical relevance.

#### Sperm origin

The use of ejaculated sperm was higher in recipients than in donors, that more frequently needed semen from a bank, PESA, or TESA (16.7 vs. 7.7, *p*<000.1). This reflects the reason for performing IVF in donors, as almost 50% of infertility causes in this group are associated with male causes, and so this data is consistent with the higher use of sperm from a bank, PESA or TESA extraction than in recipients.

#### Endometrial role

An OD program allows an excellent opportunity to evaluate uterine factors since factors affecting the ovary (donor) are separated and distinct from endometrial events in the recipients; this clinical paradigm has served as a critical tool to study both ovarian and endometrial factors contributing to implantation. In our study, donors and recipients had similar implantation rates and clinical pregnancy and live birth rates per transfer. However, it was observed a statistical difference regarding endometrial thickness between groups (seen in Table 2), but without clinical relevance, showing that uterus aging doesn’t seem to influence pregnancy rates at recipients if endometrial thicknesses were adequate. According to a recent publication, the delivery rate after egg donation was slightly affected by the age of the recipient, and a decline in delivery rates compared with younger women was only seen after the recipient is ≥44 years old (*p*=0.001,95% CI -3.06 to 9.49%) (Zegers-Hochschild *et al*., 2021).

### Limitation of the study

The data used were extracted from medical records and contained information filled in by several doctors and embryologists over the fifteen years of treatments; the lack of standardization in the filling is a measurement bias. Although this is the largest study in Brazil, the number of cycles is small compared to other countries and studies. By only evaluating outcomes in fresh cycles, we could not assess cumulative pregnancy rates.

## CONCLUSION

This study demonstrated that laboratory evolution is similar between oocyte recipients and donors. Moreover, it was observed that demographic and clinical characteristics have a secondary role in oocyte donors under 35 years and recipients without comorbidities under 50 years and were not associated with pregnancy outcomes, emphasizing the power of oocyte quality on the success of ICSI treatment. Although the OD treatment presents numerous clinical challenges, including the synchronization of donor/recipient cycles - the optimization of ovarian stimulation for donors, and the successful preparation of the endometrium in recipients- similar results between donors and recipients reinforce that an OD program, that offers good and comparable results, is fair and worth being motivated. Disseminating this information is important to encourage women to reflect on altruistic motivations for OD and promote female solidarity across different generations.
